# Neutralizing Antibodies and Pathogenesis of Hepatitis C Virus Infection

**DOI:** 10.3390/v4102016

**Published:** 2012-10-09

**Authors:** Samira Fafi-Kremer, Catherine Fauvelle, Daniel J. Felmlee, Mirjam B. Zeisel, Quentin Lepiller, Isabel Fofana, Laura Heydmann, Françoise Stoll-Keller, Thomas F. Baumert

**Affiliations:** 1 Inserm, U748, Strasbourg, France ; Email: samira.fafi-kremer@unistra.fr (S.F.-K.); catherine.fauvelle@etu.unistra.fr (C.F.); felmlee@unistra.fr (D.J.F.); mirjam.zeisel@unistra.fr (M.B.Z.); lepiller@unistra.fr (Q.L.); isabel.fofana@unistra.fr (I.F.); heydmann@unistra.fr (L.H.); francoise.stoll@unistra.fr (F.S.-K.); 2 Université de Strasbourg, Strasbourg, France; 3 Laboratoire de Virologie, Hôpitaux Universitaires de Strasbourg, Strasbourg, France; 4 Pôle Hépato-digestif, Hôpitaux Universitaires de Strasbourg, Strasbourg, France

**Keywords:** antiviral, evasion, liver, transplantation, vaccine

## Abstract

Hepatitis C virus (HCV) infection is a major cause of chronic liver disease worldwide. The interplay between the virus and host innate and adaptive immune responses determines the outcome of infection. There is increasing evidence that host neutralizing responses play a relevant role in the resulting pathogenesis. Furthermore, viral evasion from host neutralizing antibodies has been revealed to be an important contributor in leading both to viral persistence in acute liver graft infection following liver transplantation, and to chronic viral infection. The development of novel model systems to study HCV entry and neutralization has allowed a detailed understanding of the molecular mechanisms of virus-host interactions during antibody-mediated neutralization. The understanding of these mechanisms will ultimately contribute to the development of novel antiviral preventive strategies for liver graft infection and an urgently needed vaccine. This review summarizes recent concepts of the role of neutralizing antibodies in viral clearance and protection, and highlights consequences of viral escape from neutralizing antibodies in the pathogenesis of HCV infection.

## 1. Introduction

Hepatitis C virus (HCV) infection is a leading contributor to global chronic liver disease. About 160 million people are currently infected with HCV (~ 3% of the world's population), and HCV is a cause of a third of all deaths from cirrhosis and hepatocellular carcinoma (HCC) [1]. Initial HCV infection is most often followed by chronic hepatitis with persistence of viremia in up to 85% of individuals [2]. Chronic HCV infection is characterized by variable degrees of hepatic inflammation and fibrosis with an increased risk of progression to cirrhosis and HCC [2]. HCV-related cirrhosis and HCC are leading indications for liver transplantation (LT), representing 20-30% of adult elective liver transplants performed in Europe and North America [3]. Upon initial infection of the chimpanzee model, HCV RNA levels increase rapidly with a mean doubling time of 12 hours [4]. Thereafter, the viremia increase slows significantly. The innate response of hepatocytes, including interferon I responses, plasmacytoid dendritic cells, and natural killer (NK) cells, contributes to the second phase of slowed viral replication with a mean doubling time of 7.5 days [5]. HCV infection results in a robust innate immunity response including induction of several interferon stimulated genes (ISGs) [6,7]. However, through its structural and non-structural proteins, HCV interferes with innate immunity signaling pathways by interacting with factors that regulate ISGs (core, NS3/NS4A, NS5A), hence attenuating innate responses [8,9,10].

Aside from mechanisms to escape innate immunity, HCV also employs strategies to escape host adaptive immunity. Adaptive immune responses are mediated by the humoral and cellular immune systems. HCV-specific T lymphocytes are detectable 5 to 9 weeks after infection [11,12] which coincides with the onset of hepatitis. Both CD4+ and CD8+ T cells have been shown to play major roles in the outcome of HCV infection. CD8+ T cells inhibit HCV replication by cytolytic and non-cytolytic effector mechanisms [13], which are highly dependent on sufficient CD4+ T-cell cooperation. Indeed, vigorous peripheral and intrahepatic virus-specific T cell responses that target multiple epitopes have been described in patients who recover from HCV infection [5,11]. Consistent with these findings, a weak and functionally impaired T cell response was reported in patients who fail to clear the virus [13]. Viral escape, lack of CD4+ T-cell cooperation, and regulatory T cell suppression are all factors that potentially contribute to exhausting T cell responses in chronically infected patients [13]. 

In contrast to T cell responses, humoral responses have long been thought to play a marginal role in the outcome of HCV infection [14]. Unfortunately, studies of host neutralizing responses against HCV have historically been hampered by the lack of a convenient tissue culture system for HCV entry and infection. However, within the last decade, the development of model systems enabling study of HCV entry has significantly advanced our understanding of viral entry and escape. These models have reenergized interest in the role of neutralizing antibodies (nAbs) in the pathogenesis of liver disease and their potential in development of antivirals and vaccines.

In this review, we will highlight recent concepts and findings that have revealed the importance of nAbs in HCV clearance and their impact on HCV pathogenesis. Furthermore, we will describe mechanisms of viral escape from nAbs.

## 2. Neutralizing antibodies and envelope glycoproteins - a moving target

The antibody response to HCV *in vivo* is directed against several viral proteins [14]. However, nAbs that block HCV entry are specifically directed toward the viral envelope, particularly envelope glycoprotein E2. Although the crystal structure of E1-E2 has not been solved, recent findings based on molecular and biochemical analyses provide key information on the structural organization and antigenic determinants of E1 and E2 envelope glycoproteins [15]. The envelope glycoproteins E1 and E2 are type I transmembrane proteins with an N-terminal ectodomain and a short C-terminal transmembrane domain (TMD). The N-terminal ectodomains of E1 and E2 are heavily glycosylated and the glycans are thought to play major roles in E1-E2 folding, HCV entry, and neutralization [16]. Virion-associated E1 and E2 envelope glycoproteins form large covalent complexes stabilized by disulfide bridges [17], forming a functional glycoprotein that mediates viral entry into host cells [17]. 

Initial HCV attachment to the cell surface is likely facilitated by interaction with attachment factors like glycosaminoglycans [18,19] and probably low-density lipoprotein (LDL) receptor [20], though this internalization pathway might not lead to sustained viral infection [21,22]. Upon initial attachment, at least six host entry factors are important for particle internalization. These include scavenger receptor class B type 1 (SR-BI), CD81, the tight junction proteins claudin 1 (CLDN1) and occludin (OCLN) [23], the receptor tyrosine kinases [24] and the Niemann-Pick C1-like 1 cholesterol absorption receptor [25]. Functional analysis and neutralization experiments using sera from chronically HCV infected patients have demonstrated that host neutralizing responses target viral entry at a step after initial HCV binding; most likely by interrupting HCV E2-CD81 or HCV E2-SR-BI interactions, or by inhibiting membrane fusion [26]. Indeed, several E2 domains have been shown to play pivotal roles in viral entry and neutralization. Two regions in the E2 envelope glycoprotein have increased genetic variability within a quasispecies and among genotypes and have therefore been identified as hypervariable regions (HVR). The first 27 amino acids of E2 compose the first HVR (HVR1), which plays an important role in viral fitness, likely due to the involvement of SR-BI-mediated entry [27], assembly, and release of virus particles [28], as well as the HCV membrane fusion process [28]. Although HVR1 is a prime target for neutralizing antibodies, the antibodies that target HVR1 exhibit poor cross-neutralization potency across different HCV isolates due to the region’s high variability [29]. Both deletion of HVR1 and insertion of single mutations in this region significantly increase sensitivity of HCV variants for neutralization by monoclonal antibodies (mAbs) or patient-derived sera [17,30]. Antibodies that demonstrate broadly neutralizing activity tend to be directed against conserved and conformational epitopes within E2 and most often inhibit the interaction between CD81 and E2 [31,32,33,34,35,36,37,38,39,40]. The region located immediately downstream of HVR1 has been shown to contain a potent and highly conserved epitope [41]. This epitope is defined by the mouse mAb AP33 and the rat mAb 3/11. These antibodies inhibit interactions between E2 and either CD81 [34] or heparan sulfate [42]. Recently, conformational and widely conserved epitopes were identified in E1 and E2 [38,43,44,45]. The human mAb AR3, which defines one of these epitopes (aa 396–424; 436–447; 523–540), neutralizes genetically diverse HCV isolates and protects against challenge of heterologous HCV quasispecies in a human liver–chimeric mouse model [38]. Similar antibodies recognizing conformational epitopes, in this case defined by the mouse mAb D32.10 (aa 297–306; 480–494 and 613–621), were observed to circulate at high levels in the sera of patients with resolved HCV infection [44,45].

Recently, epitopes have been identified in the E2 protein at residues 412–426 (epitope I) and 434–446 (epitope II). Interestingly, antibodies that bind one of these epitopes may be interfering antibodies, in that the binding of a non-neutralizing antibody to epitope II disrupts virus neutralization mediated by an antibody binding at epitope I [46,47]. However, discrepant data were reported recently by Tarr *et al.*, which indicate that human antibodies that target the aa 434–446 region do not inhibit neutralization but instead are capable of neutralizing HCV pseudoparticles (HCVpp) and cell-culture derived HCV (HCVcc) entry [48].

## 3. Humoral responses contribute to control and protection against HCV infection.

It has long been known that humoral responses including the generation of neutralizing antibodies are key protective strategies of host immunity. Farci *et al.* first described neutralizing anti-HCV antibodies in chimpanzees [49]. These antibodies targeted epitopes within the HVR1 of envelope glycoprotein E2 [49]. Interestingly, immunization of chimpanzees with a rabbit serum directed against a synthetic HVR1 peptide protected these animals from viral variants displaying the same HVR1 but not against other viral variants [50]. 

Two years later, Feray *et al.* studied retrospectively a cohort of patients who underwent LT due to hepatitis B virus- (HBV) (210/428) or HCV-induced (218/428) liver cirrhosis. Anti-HBV immunoglobulins, which may have incidentally been contaminated with anti-HCV immunoglobulins, were administered to 46 of 218 of the previously HCV-infected patients. Following transplantation, incidence of HCV viremia was lower in patients receiving immunotherapy (54%) than in patients whose therapy did not include anti-HCV antibodies (94%) [51]. Moreover, among the 210 patients who were HCV-negative before LT, acquired HCV infection was less frequently observed in those patients who received immunotherapy than those who did not (26% *vs.* 47%). 

These initial observations indicate the potential contribution of neutralizing antibodies in viral control. Subsequently, the use of HCVpp bearing envelope glycoproteins of different genotypes or different patient isolates has allowed more thorough characterization of the role of neutralizing antibodies in both spontaneous control of infection and protection against re-infection. 

### 3.1. Spontaneous control of HCV infection by humoral immune responses.

The majority of HCV infections evolve to chronicity; however, a minority of individuals (25–30%) spontaneously clear HCV infection. Early studies on the role of nAbs in this phenomenon generated controversial results. To address whether nAb activity associates with immune control of HCV infection and viral clearance, Logvinoff et al. retrospectively studied the presence of nAbs in plasma samples from seven health care workers who were infected with HCV genotype 1 by needlestick exposure. This early study apparently indicated that there may not be a major role for nAbs in spontaneous control of infection since nAbs were detected in two out of seven acutely infected patients and nAb presence was not associated with viral clearance [52]. Netski *et al.* reported similar results by examining 12 intravenous drug users whose serum contained only low titers of nAbs restricted to the IgG1 subclass [53]. However, these studies were highly limited by the fact that the viral surrogate ligand was derived from a different isolate than the virus present in the infected patient, thus precluding the detection of isolate-specific antibodies. Later, studies using well-defined nosocomial or single-source HCV outbreaks with a defined inoculum enabled researchers to study the role of isolate-specific nAbs in the control of HCV infection in humans. Using the HCVpp model system, two studies have demonstrated that nAbs are induced in the early phase of infection in patients who subsequently clear the virus [54], or control viral infection [55].

Lavillette *et al.* studied the kinetics of humoral responses during the acute phase of HCV infection in 17 patients, all of whom had been accidentally infected with the same viral inoculum in a hemodialysis center. All patients were followed-up for six months [55]. While anti-HCV antibodies were detected in all 17 patients, only a fraction of patients (group 1) were characterized by a progressive decrease in viremia finally resulting in a resolution of infection, while this decrease was not observed in other patients (group 2). *In vitro* neutralization assays demonstrated that only the sera derived from patients of group 1 were able to neutralize HCVpp. These data indicate a correlation between the presence of nAbs during the acute phase of infection and a reduction in viremia [55]. 

This hypothesis was confirmed and extended by the study of Pestka *et al.* [54], who studied sera from a cohort of pregnant women whom had been accidentally infected with a single HCV genotype 1b inoculum after administration of HCV-contaminated anti-D immunoglobulins in 1978–1979 [54]. Follow-up over 17 years and the use of the HCVpp model system demonstrated that viral clearance was associated with the presence of nAbs during the acute phase of infection. The quantity and efficacy of circulating nAbs diminished over time following viral clearance. In contrast, little to no levels of nAbs were detected during the acute phase in women progressing to chronic infection. Significant antibody titers were subsequently detected in these individuals 10 to 17 years after viral inoculation. These data indicate that a strong and early production of nAbs may contribute to control of the virus during the acute phase of HCV infection and facilitate viral elimination by cellular immune responses. These findings were later corroborated by Dowd *et al.* who demonstrated that a high concentration of nAbs coincided with viral clearance in individuals who spontaneously resolved HCV infection [56]. Most recently, a detailed immunological study of a single case of spontaneous recovery from chronic HCV infection confirmed the important role of nAbs in HCV clearance [57].

### 3.2. Prevention of HCV reinfection by B cell responses.

In addition to their role in control of infection during the acute phase, nAbs appear to also protect from reinfection. During a monthly follow-up of a high-risk of reinfection group of 22 intravenous drug users who had previously spontaneously cleared HCV infection, 11 individuals were observed to reacquire HCV infection [58]. Among these patients, 83% again efficiently spontaneously cleared viral infection. Moreover, the secondary infection in these patients was characterized by reduced viremia relative to the primary infection. *In vitro* neutralization assays allowed detection of cross-neutralizing antibodies in the sera of 60% of patients who spontaneously cleared infection after reexposure to HCV, while less cross-neutralizing antibodies were detected in the sera of patients who progressed into chronic infection. Spontaneous clearance of HCV reinfection may thus be associated with production of cross-neutralizing humoral responses that are able to decrease or even control viremia (in magnitude and duration) [58]. However, an important caveat in this analysis is the fact that nAbs are not the sole factors involved in protection against reinfection, and this population is likely genetically predisposed to be able to clear HCV infection. Indeed, a recent study on chimpanzees has demonstrated that a complex network of innate and adaptive immune responses orchestrates protection against reinfection [59].

## 4. Implications of viral evasion from neutralizing antibodies in pathogenesis of HCV infection.

HCV is able to induce protective nAbs *in vivo*, yet the reasons why the majority of patients progress to chronic hepatitis are not well-characterized. A major contributor to chronic HCV infection includes the high variability of HCV. Indeed, the virus circulates in the patient as constantly and rapidly evolving genetically distinct, but closely related, variants within the quasispecies. The simultaneous presence of different variants allows the rapid selection of mutants which are best adapted to the host environmental changes, hence the virus could persist in the body despite the presence of neutralizing antibodies. HCV persists via several evasion strategies acting both in the context of chronic infection and in the liver transplant setting.

### 4.1. HCV escape and persistence in chronic HCV infection.

Genetic analysis of the quasispecies variation among patients has demonstrated a correlation between viral clearance and a slowly adapting population. An evolutionarily stable population results in a decreased diversity of HCV variants. On the other hand, chronicity is associated with rapid evolution of the quasispecies, with a multiplicity of variants [60]. Farci *et al.* demonstrated that the patterns of progression to chronicity were mostly related to the selective pressure put upon the HVR1 region of HCV E2 [60].

The link between viral quasispecies evolution, nAbs, and progression to chronicity has later been confirmed in a chronic HCV patient monitored over a 26-year period [61]. Using the HCVpp system, von Hahn *et al*. were able to analyze the envelope glycoproteins E1-E2 of quasispecies circulating in this patient at serial time points over 26 years. Neutralization assays using autologous and heterologous sera confirmed that HCV continuously escapes the host’s immune system by repeated mutational changes. Generating these mutants results in loss of recognition of the HCV envelope glycoproteins by nAbs [61]. HCV persists by always being a step ahead of host humoral immunity, observed by a lag of the nAb response behind the rapidly evolving HCV envelope glycoprotein sequences of the quasispecies population. Consistent with this, a study of eight patients monitored throughout acute infection revealed the same phenomenon in the early stages of infection. Moreover, patients who progressed to chronic infection present low titer of nAbs during early acute phase [56].

**Figure 1:**
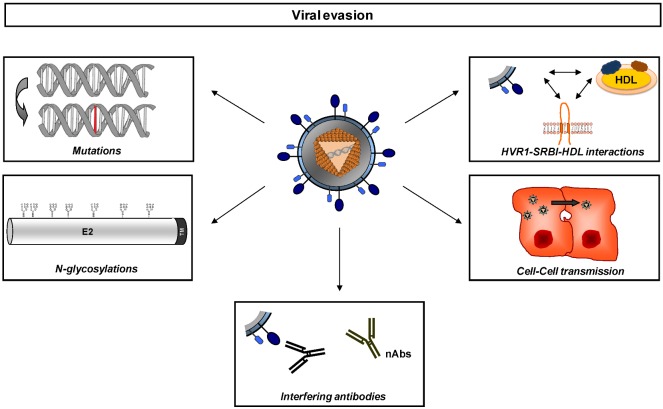
Strategies of viral evasion during Hepatitis C virus (HCV) infection. HCV has adapted multiple mechanisms to escape host immunity. The low-fidelity HCV RNA polymerase NS5B introduces point mutations to generate a genetic diversity within the quasispecies in an infected host. Many of the viable variants cluster in high-variability regions (e.g., HVR1) of the glycoprotein sequences, which can contribute to differential binding and usage of HDL and SR-BI of different variants, providing flexibility for adaptation against host humoral immunity. Glycosylation sites conceal important functional domains of E2 by forming a glycan shield that reduces the viral immunogenicity and the access of the epitopes to nAbs (reviewed in [68]). Additionally, the action of nAbs may also be disturbed by the presence of interfering antibodies. Another way of escaping neutralizing antibodies is by avoiding the circulation altogether, by dissemination via cell-to-cell transmission. *HDL: High Density Lipoprotein ; HVR1: Hypervariable Region 1; nAbs: neutralizing Antibodies*

The high level of genetic variability of HCV is able to confer a remarkable viral fitness and adaptation to the host environment through a variety of mechanisms (Figure 1 and reviewed in [62]). HCV genetic evolution can result in single point mutations, glycosylation site modifications [63], and conformational changes that could mask envelope glycoprotein binding sites [64,65,66,67]. 

Lipoproteins may also provide protection to HCV from nAbs, either by masking viral epitopes (by associating with LDL and VLDL) or by accelerating viral entry (HDL), hence limiting exposure of viral epitopes to nAbs [30,62]. Evidence suggests that, at least in genotype 2a variants, those viruses that associate with LDL and VLDL, and are consequently distributed in low-density fractions, are more capable of escape from neutralizing antibodies than higher density viruses [69,70]. In addition to these evasion mechanisms, HCV dissemination by cell-to-cell transmission may also contribute to viral persistence by avoiding surveillance of neutralizing antibodies present in the serum [71,72]. Moreover, HCV may directly infect B lymphocytes and induce hypermutations in the heavy chains of immunoglobulins. These hypermutations may decrease the affinity and the specificity of the anti-HCV antibodies allowing the virus to escape from the immune system [73]. Finally, though not a viral strategy, the neutralizing activity of nAbs may incidentally be blocked by interfering antibodies, as previously mentioned [46,47].

### 4.2. Escape from B cell responses are a key determinant for liver graft infection.

HCV escape from nAbs plays a key role not only in viral persistence during chronic HCV infection, but also in *de novo* infection of the liver graft during LT [74]. Infection of the liver graft occurs within a few hours of graft reperfusion despite the presence of anti-HCV antibodies. Among the diversity of circulating viral variants before transplantation, only a fraction of them persist post-transplantation [75,76]. The grafting of the new liver and coinciding initiation of immunosuppressive therapy acts to generate a bottleneck effect that selects the most efficient variants capable of infecting the hepatocytes of the liver graft [74]. Functional analysis using patient-derived HCVpp demonstrated that those HCV variants that infect the liver graft (selected variants) were characterized by markedly enhanced entry compared to variants not present in the post-transplantation setting. Interestingly, persistent variants were only poorly neutralized by antibodies present in pre-transplant serum compared to variants not detected post-transplantation that were efficiently neutralized [74]. These results suggest that escape from neutralizing antibodies and efficient entry into hepatocytes play a major role in reinfection of the liver graft. Interestingly, reverse genetic studies and functional analyses on patient-derived HCVpp and HCVcc identified a novel mechanism of viral evasion during liver transplantation where co-evolution simultaneously occurs between cellular entry factor usage and escape from neutralization. In this study, one of these selected variants harbors mutations (F447, S458 and R478) that modulate CD81-dependency of HCV entry, alter E2-CD81 interaction, and mediate escape from antibodies at post-binding steps closely related to HCV-CD81 interactions [77]. Additionally, this study used a large panel of patient-derived antibodies, which demonstrates the functional impact of these mutations in viral persistence in chronic HCV infection [77].

## 5. Conclusions and perspectives

Significant advances have been achieved during the last decade to elucidate the molecular mechanisms of HCV entry and neutralization. The knowledge gained by these studies has renewed enthusiasm in the therapeutic potential of neutralizing antibodies against HCV infection. This potential is bolstered by recent findings that a prime-boost strategy using virus-like particles, pseudotyped for HCV proteins, will trigger broadly neutralizing antibodies in macaques [78]. Furthermore, mAbs can protect against heterologous HCV quasispecies in a human liver–chimeric mouse model, providing evidence that broadly neutralizing antibodies against HCV can prevent heterologous HCV infection [38,43,79]. These studies suggest that the development of a prophylactic vaccine against HCV is likely achievable. The resolution of the structure of the HCV envelope glycoproteins and information garnered from high-throughput neutralization screening approaches would contribute in identifying novel viral targets, which in turn promise the development of efficient vaccine strategies. 
